# Magnetic Nanoparticles Conjugated with Peptides Derived from Monocyte Chemoattractant Protein-1 as a Tool for Targeting Atherosclerosis

**DOI:** 10.3390/pharmaceutics10020062

**Published:** 2018-05-24

**Authors:** Chung-Wei Kao, Po-Ting Wu, Mei-Yi Liao, I-Ju Chung, Kai-Chien Yang, Wen-Yih Isaac Tseng, Jiashing Yu

**Affiliations:** 1 Department of Chemical Engineering, National Taiwan University, Taipei 10617, Taiwan; r04524101@ntu.edu.tw (C.-W.K.); r05524112@ntu.edu.tw (P.-T.W.); 2 Department of Applied Chemistry, National Pingtung University, Pingtung 90003, Taiwan; myliao@mail.nptu.edu.tw; 3 Department and Graduate Institute of Pharmacology College of Medicine, National Taiwan University, Taipei 10617, Taiwan; r05458006@ntu.edu.tw; 4 Institute of Medical Device and Imaging, National Taiwan University, Taipei 10617, Taiwan; 5 Molecular Imaging Center, National Taiwan University, Taipei 10617, Taiwan

**Keywords:** iron oxide magnetic nanoparticle, monocytes, MCP-1, atherosclerosis

## Abstract

Atherosclerosis is a multifactorial inflammatory disease that may progress silently for long period, and it is also widely accepted as the main cause of cardiovascular diseases. To prevent atherosclerotic plaques from generating, imaging early molecular markers and quantifying the extent of disease progression are desired. During inflammation, circulating monocytes leave the bloodstream and migrate into incipient lipid accumulation in the artery wall, following conditioning by local growth factors and proinflammatory cytokines; therefore, monocyte accumulation in the arterial wall can be observed in fatty streaks, rupture-prone plaques, and experimental atherosclerosis. In this work, we synthesized monocyte-targeting iron oxide magnetic nanoparticles (MNPs), which were incorporated with the peptides derived from the chemokine receptor C-C chemokine receptor type 2 (CCR2)-binding motif of monocytes chemoattractant protein-1 (MCP-1) as a diagnostic tool for potential atherosclerosis. MCP-1-motif MNPs co-localized with monocytes in in vitro fluorescence imaging. In addition, with MNPs injection in ApoE knockout mice (ApoE KO mice), the well-characterized animal model of atherosclerosis, MNPs were found in specific organs or regions which had monocytes accumulation, especially the aorta of atherosclerosis model mice, through in vivo imaging system (IVIS) imaging and magnetic resonance imaging (MRI). We also performed Oil Red O staining and Prussian Blue staining to confirm the co-localization of MCP-1-motif MNPs and atherosclerosis. The results showed the promising potential of MCP-1-motif MNPs as a diagnostic agent of atherosclerosis.

## 1. Introduction

Atherosclerosis, the primary cause of cardiovascular diseases, is a chronic inflammatory disorder in the walls of large arteries or the medium and intima of large arteries. Inflammation is the immune system’s response to injury and has been implicated in the pathogeneses of aortas. After lipid-rich plaques and cholesterol particles accumulate within the artery wall, endothelial cell dysfunction/activation is then triggered by the accumulation of low-density lipoprotein (LDL) and other lipoproteins. Afterward, the inflamed endothelial cells and oxidized lipids induce the excretion of chemokines, cytokines, and mediators of inflammation into the bloodstream for monocytes and other immune cells recruitment to the site. As the monocytes migrate into the aorta wall, they differentiate into dendritic cells, macrophages, or foam cells [1,2]. At the same time, the continued recruitment and accumulation of leukocytes is associated with the development of vulnerable plaques. The plaques can become unstable and thus rupture, leading to thrombosis, myocardial infarction, or stroke. In addition, the invasion and accumulation of white blood cells create atheromatous plaques, which make the artery walls lose their flexibility and obstruct blood circulation [3,4]. Also, the inflammation hypothesis has recently been proved by genetic evidence. The latest research has shown the relation between coronary artery disease and the transendothelial pathway by genetic evidence [5,6].

Chemokines play important roles in atherosclerotic vascular disease. They are also expressed by cells of the vessel wall [7]. As endothelial cells undergo inflammatory activation, the increased expression of cell adhesion molecules, such as vascular cell adhesion molecule-1 and intercellular adhesion molecule-1, promotes the adherence of monocytes. Furthermore, the monocytes migration is controlled by the concentration gradient of monocytes chemoattractant protein-1, a chemokine that binds to the C-C chemokine receptor named CCR2. Then, monocytes transmigrate into the innermost layer of the arterial wall, pass between the endothelial cells to differentiate into macrophages, and transform into foam cells [8,9].

In atherosclerotic arteries and atheromatous plaques, MCP-1 can be found in endothelial cells, macrophages, and vascular smooth muscle cells. MCP-1 recruits monocytes into the subendothelial cell layer and thus advances the development of atherosclerosis [10,11].

Due to the internal filters in the human body such as the liver, kidneys, and lymph nodes, site-specific delivery by the conjugation of the modification of ligands can provide stable routes to avoid damaging normal tissue and enhance therapeutic efficiency. Pan et al. developed VCAM-1-targeting nanocarriers with a four-fold aggregation in the aortas of atherosclerosis model mice compared to control model mice [12]. Chung et al. developed peptide amphiphile micelles incorporated with the chemokine receptor CCR2-binding motif of MCP-1 for atherosclerosis targeting. The results showed that MCP-1 peptide amphiphile micelles (PAMs) bind with monocytes in vitro and can be detected in early-stage atherosclerotic aortas [13].

Nanoparticles have been used for various applications in the biomedical field. The increasing permeability allows more small sized particles to migrate into the intimal layer, resulting in enhanced permeability and retention (EPR) effect [14,15]. When atherosclerosis lesions develop, endothelial cells are prone to be leaky and fragile, which enhances the EPR effect.

Iron oxide magnetic nanoparticles possess superior physical and chemical properties, such as superparamagnetism and the quantum tunneling of magnetization. Additionally, iron oxide magnetic nanoparticles (MNPs) are also non-toxic, biocompatible, and easy to separate under external magnetic fields. Owing to their unique properties, such as superparamagnetism, high surface area, large surface-to-volume ratio, low toxicity, and easy separation under external magnetic fields, iron oxide MNPs have enormous potential in fields such as magnetic resonance imaging (MRI), bioseparation, environmental treatment, fluorescence labeling, and biomedical and bioengineering usage [16,17].

Although Fe_3_O_4_ MNP is a promising drug carrier, there are still drawbacks to its use, such as aggregation and oxidation to γ-Fe_2_O_3_. Therefore, polymer coatings are usually applied to modify its surface characteristics [18]. In recent years, the common reagents employed for the modification of iron oxide MNPs have included surfactants, polymers, and natural dispersants [19,20,21,22]. Nevertheless, the most used modification for medical applications is dextran, a biocompatible derivative. In aqueous solutions, dextran interacts with metals and covers its surface, yielding aggregates with hydrodynamic diameters between 20 nm and 150 nm [23,24].

In this work, iron oxide MNPs and MCP-1-motif iron oxide MNPs were stained with Cyanine 5 (Cy5) in fluorescence microscopy. The properties of MNPs were characterized. For in vitro cell experiments, cell viability was measured by the MTT assay and Live/Dead staining. To test the spatial distribution of cells and MCP-1-motif iron oxide MNPs, two types of cells, including 3T3 cells and WEHI 274.1 monocytes [13], were cultivated with iron oxide MNPs.

Apolipoprotein E-knockout (ApoE KO) mice, which are the most commonly used and well-characterized animal model of atherosclerosis, were treated with a high-fat diet for further plaque development and the monocytes accumulation was continuous and proportional to disease progression [25,26,27].

**Figure 1:**
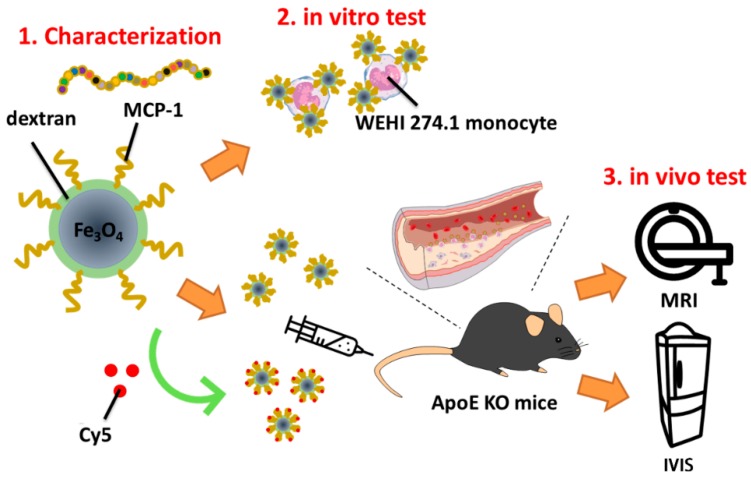
Scheme of the experiment.

Through the in vivo test, ApoE KO mice fed a high-fat diet and C57BL/6 wild-type mice fed a normal diet for four weeks made up the atherosclerosis model and control group, respectively. Mice were injected with iron oxide MNPs through the tail vein and the nanoparticle distribution was observed by magnetic resonance imaging (MRI) and in vivo imaging system (IVIS). Figure 1 gives a representative scheme of this research.

## 2. Materials and Methods

### 2.1. Materials

MCP-1 peptides (YNFTNRKISVQRLASYRRITSSK) were purchased from Yao-Hong Biotechnology (New Taipei, Taiwan). Iron oxide nanoparticles and the conjugation of MCP-1 peptides and iron nanoparticles were obtained from MagQu (Taipei, Taiwan). 3T3 cells and WEHI 274.1 monocytes were purchased from American Type Culture Collection (ATCC). Cyanine 5 NHS Ester were purchased from Lumiprobe (Hunt Valley, MD, USA). Dulbecco’s modified Eagle’s medium-high glucose (DMEM-HG), 2-mercaptoethanol (β-ME), thiazolyl blue tetrazolium bromide (MTT solvent), and Oil Red O were purchased from Thermo (Waltham, MA, USA). Fetal bovine serum (FBS) and antibiotic antimycotic solution (penicillin/streptomycin/amphotericinβ) were purchased from Biological (Cromwell, CT, USA).

### 2.2. Characterization of Iron Oxide MNPs

The structure of iron oxide MNPs were characterized by scanning electron microscopy (SEM) (J NanoSEM 230, Nova, Pallini, Greece) and transmission electron microscopy (TEM) (H-7650, Hitachi, Tokyo, Japan). Particle size and zeta potential were measured in a Zetasizer nanosystem (Zetasizer Nano, Malvern, UK). Iron oxide MNPs solution was stored at −20 °C overnight and then moved to a freeze dryer overnight to remove all water. The iron oxide MNPs powders were investigated by X-ray photoelectron spectroscopy (XPS) (Theta Probe, Thermo Scientific, Waltham, MA, USA) for composition synthesis (carbon, nitrogen, and oxygen) and by a magnetometer (MPMS7, Quantum Design, San Diego, CA, USA) for magnetic hysteresis loop.

### 2.3. Cell Culture

WEHI 274.1 monocytes (ATCC, Manassas, VA, USA) were cultured in DMEM-HG culture medium supplemented with 10% fetal bovine serum, 1% antibiotic-antimycotic solution, and 0.05 mM 2-mercaptoethanol for in vitro testing. The cells were cultured on a T75 flask at 37 °C in a humidified incubator under 5% CO_2_. After two days of cultivation, the medium with suspended monocytes was moved to a centrifuge tube and centrifuged at 100 relative centrifugal force (rcf) for 5 min. Then the supernatant was removed to eliminate the wastes and the monocytes were resuspended in culture medium. To estimate the number of the cells, trypan blue was used to mark the dead cells and hemocytometer was used to evaluate the number of viable cells. The suspended cells were prepared for the use of the following experiments.

3T3 cells were cultured in DMEM-HG culture medium with 10% FBS and 1% antibiotic-antimycotic solution for in vitro testing. First, the cells were seeded on a 10-cm culture dish at 37 °C in a humidified incubator under 5% CO_2_. After washing with PBS, trypsin-EDTA was added and incubated with cells for 4 min at 37 °C to detach cells from the culture plate. Then, culture medium was added to the dish. The medium with suspended cells was centrifuged at 100 rcf for 5 min. Then the supernatant was removed and the cells were resuspended in culture medium. To estimate the number of the cells, trypan blue was used to mark the dead cells and hemocytometer was used to evaluate the number of viable cells. The suspended cells were prepared for the use of the following experiments.

### 2.4. Cell Viability Evaluation

#### 2.4.1. MTT Assay

3T3 cells were cultivated with different concentrations (0.1, 0.2, and 0.3 mg Fe/mL culture medium) of iron oxide MNPs for four days. Cell viability was investigated on days 1 and 4 via MTT assay. Prior to use, MTT stock solution was diluted to 0.5 mg/mL with cell culture medium. After removing the original medium and PBS buffer washing, 500 μL MTT working solution was added to each well and the mixture was placed in an incubator for 3 h. Finally, the MTT solution was replaced with the same volume of DMSO and the mixture was shaken for 30 min. The absorbance value of the product solution was observed at 570 nm. The whole process was operated without light exposure.

#### 2.4.2. Live/Dead Assay

After WEHI 274.1 monocytes and 3T3 cells were cultivated with different concentrations (0.1, 0.2, and 0.3 mg Fe/mL culture medium) of iron oxide MNPs for four days, the samples were stained by Live/Dead dye to check cell viability. Live cells were stained fluorescent green due to reveal intracellular esterase activity that deacetylated fluorescein diacetate to a green fluorescent product. Dead cells were stained fluorescent red, as their compromised membranes were permeable to nucleic acid stain (propidium iodide). Photos were taken by an inverted fluorescence microscope (Olympus, IX-71, Tokyo, Japan).

### 2.5. In Vitro Imaging of Nanoparticles

First of all, MCP-1-motif iron oxide MNPs were stained with Cy5 fluorescence and the composition of the reaction was as follows: 2 μL MCP-1-motif iron oxide MNPs solution (8.2 mg Fe/mL), 10 μL Cy5 solution (1 mg/mL), and 90 μL sodium bicarbonate solution (0.1 M, pH 8.3). The reaction occurred at 4 °C refrigerator overnight with shaking. After the reaction, the solution was centrifuged 110 rcf for 10 min and the supernatant was removed in order to remove excess Cy5 fluorescence. The process was repeated twice to avoid excess Cy5 reacting with cells. The Cy5-MCP-1-motif iron oxide MNPs were refilled in PBS for future use.

Thirty thousand monocytes were cultured in 48-well plates and incubated with Cy5-MCP-1-motif iron oxide MNPs suspension for 1 h at 37 °C in a humidified incubator under 5% CO_2_. Finally, DAPI was used for nucleus staining and the results were achieved with an inverted fluorescence microscope. 3T3 cells with same quantities were used for control cells.

### 2.6. Animal Model

ApoE KO mice (male, 7 weeks old) and C57BL/6 wild-type mice (male, 7 weeks old) were given a high-fat diet (HFD, 45% fat, 35% carbohydrate, 20% protein) and/or a normal diet (ND, 10% fat, 70% carbohydrate, 20% protein) in National Yang-Ming University, Taipei, Taiwan (for MRI) and Laboratory Animal Center, National Taiwan University College of Medicine, Taipei, Taiwan (for IVIS). The animal protocol was approved by National Taiwan University College of Medicine Laboratory Animal Center (#20160214) (Effective dates: 2016/07/01 ~ 2020/06/30).

The high-fat diet and gene deficiency promoted atherosclerosis plaque progression. Mice were shaved and the tail veins were dilated and sterilized with 70% ethanol before iron oxide nanoparticles injection (10 μg/g mice). The injection detail and mice choice are shown in Table 1 and Table 2.

### 2.7. Nuclear Magnetic Resonance Imaging (MRI)

All of the mice were anesthetized by ether and were measured using a T2*-contrast (axial view) FLASH sequence 7.0 T imaging (BRUKER BIOSPEC 70/30 MRI, Billerica, MA, USA) for every 2-mm sectioning thickness. (Repetition time (TR) = 200 ms, Echo time (TE) = 5 ms, Matrix 256 × 256 pixel, Field of view (FOV) = 4 × 4 cm, Flip angle = 30°) Images were acquired at 40 h after nanoparticles injection [28].

### 2.8. Non-Invasive In Vivo Imaging System (IVIS)

For IVIS, iron oxide MNPs all had been modified with Cy5 fluorescence and the imaging time spots of IVIS were 0, 2, 8, 24, 48, 72 h after nanoparticles injection [29]. Mice were anesthetized with 2.5% isoflurane in O_2_ and whole-body fluorescence imaging was conducted by IVIS. The emission at 680 nm was measured with an optimal excitation wavelength of 640 nm (FOV: 12.5, f2, 0.75 s). Mice were then euthanized via CO_2_ overdose and the aorta, heart, liver, spleen, lung, and kidney were harvested. The aorta and organs fluorescence were also conducted using IVIS. After imaging, all were immersed in 5% formaldehyde for fixation and preservation [13].

### 2.9. Histology Staining

Freshly dissected tissues were covered with enough O.C.T for a few minutes in a labeled small weigh boat. Then the prepared sample was placed in a metal beaker filled 2/3 full with isopentane and subsequently placed in a Dewar of liquid nitrogen. Samples (6–8 μm) were cryosectioned and stained with hematoxylin for 1 min, or Prussian Blue staining for iron oxide MNPs for 30 min, or Oil Red O staining for atherosclerosis for 5 min, depending on the investigation [30,31,32,33].

### 2.10. Statistical Analysis

All data are expressed as means ± standard deviation. A comparison of different groups was determined using Student’s *t*-test and significant difference was assumed at *p*-value ≤ 0.05. The statistical data was analyzed using ORIGIN^®^ 8.6 (OriginLab Corp., Northampton, MA, USA).

## 3. Results and Discussion

### 3.1. Characterization of Iron Oxide MNPs

TEM and SEM images were employed to observe the structures of iron oxide MNPs and MCP-1-motif MNPs. The SEM images reveal that most of the nanoparticles are quasi-spherical and attempt to aggregate in the solid state because of their high surface energy [34] (Figure S1). Due to peptide surface modification, peptides derived from MCP-1 might have physical, such as Van der Waals force, and chemical interactions, such as NH⋯OH-bonds, with each other and thus impede nanoparticles dispersion [35,36].

**Figure 2:**
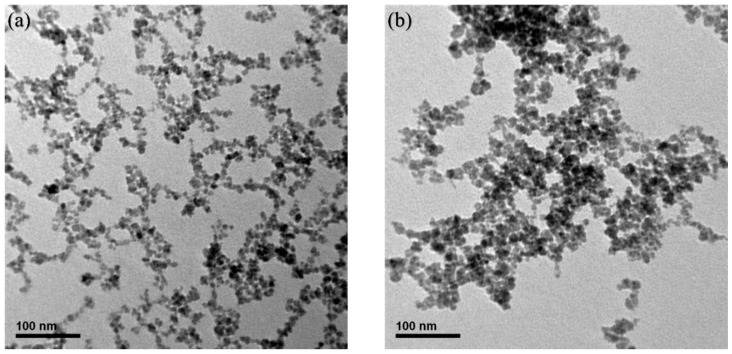
Characterization of (**a**) magnetic nanoparticles (MNPs) and (**b**) monocytes chemoattractant protein-1 (MCP-1)-motif MNPs using TEM.

Also, the TEM images show the size and the structure of the iron oxide MNPs (Figure 2). Comparing the size of two types of MNPs, MCP-1-motif MNPs formed a larger morphology and shape that is more irregular. The diameter of MCP-1-motif MNPs and MNPs were approximately 20 ± 3 nm and 10 ± 3 nm in a spherical shape, respectively.

Moreover, the hydrodynamic diameter of the iron oxide MNPs was measured by the dynamic light scattering (DLS) method to test aqueous properties (Table 3). In the results of DLS, the peaks of the diameter were approximately 90 nm and 300 nm (Figure S2). The higher value in particle size determined by the DLS method compared to the TEM image was attributed to the interaction of water molecules in the aqueous solution [37]. Furthermore, the zeta potential value of MNPs (−14.1 mV) and MCP-1-motif MNPs (−17.6 mV) were higher than −20 mV, which indicated that the dispersion was relatively stable [38]. Therefore, the nanoparticles solution would be sonicated before the experiment.

**Figure 3:**
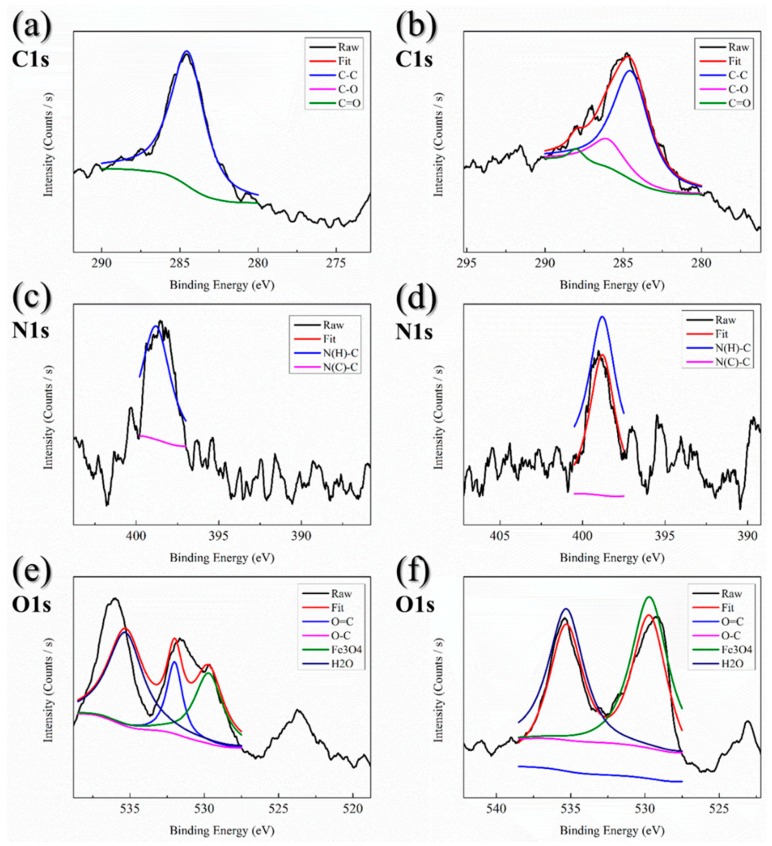
X-ray photoelectron spectroscopy (XPS) spectra of (**a**,**c**,**e**) MNPs and (**b**,**d**,**f**) MCP-1-motif MNPs.

XPS measurements were made to quantify the element composition of nanoparticles, and the results are shown in Figure 3. Three bands of the XPS survey spectrum at around 285, 397.5, and 532.5 eV represented C1s, N1s, and O1s, respectively. In Figure 3a,b, the C1s XPS spectra had a large peak at 284.5 eV, corresponding to sp^2^ hybridized carbon (C-C bonds), as well as two small peaks at 286.0 and 288.0 eV, which could be ascribed to C-O bonds and C=O bonds [39,40]. On the other hand, the N1s XPS spectra in Figure 3c,d all exhibited a major peak at around 400.0 eV. The binding energies at 398.8 and 400.0 eV, respectively, were attributed to N atoms bonded with sp^3^-hybridized C atoms (N-sp^3^C, N(H)-C bonds) and N atoms bonded with sp^2^-hybridized C atoms (N-sp^2^C, N(C)-C bonds) [41,42]. Figure 3e,f show the O1s XPS spectra of iron oxide MNPs and the two major peaks were located at around 530.0 and 535 eV. The O1s peaking at 532.0 and 533.3 eV could be assigned to oxygen in the form of O=C bonds and C-O bonds. In addition, the binding energies at 529.7 eV and 535.3 eV represented Fe_3_O_4_ and H_2_O, respectively [43,44].

Table 4 and Table S1 show the compositions of different kinds of bonds and the chemical elements of iron oxide MNPs. MNPs only had dextran shell modification, which had a large quantity of C-C bonds. Moreover, the hydroxyl bond was replaced with an amine bond, so no signal (C-O bonds) could be found in the C1s XPS spectrum of MNPs. Also, we observed that the C1s XPS spectrum of MCP-1-motif MNPs had peaks at the C-O bonds and C=O bonds because of the peptide sequence. According to the description above, the amine bond was the major composition in the MNPs consistent with the N1s XPS spectrum. On the other hand, both N(C)-C bonds and N(H)-C bonds could be found in the results of MCP-1-motif MNPs. Through the oxygen analysis, two kinds of nanoparticles presented Fe_3_O_4_ and H_2_O. With the C=O bonds existing on the shell of the nanoparticles, the ratio of Fe_3_O_4_ and H_2_O decreased compared to the control group.

Table 4 shows the chemical element composition of two iron oxide MNPs. MNPs had dextran shells, a kind of organic compound, so carbon was the major element in the composition. On the other hand, the peptides sequence and iron oxide core presented oxygen, so the ratio of oxygen was higher than those of the other two elements.

### 3.2. Magnetic Measurements

**Figure 4:**
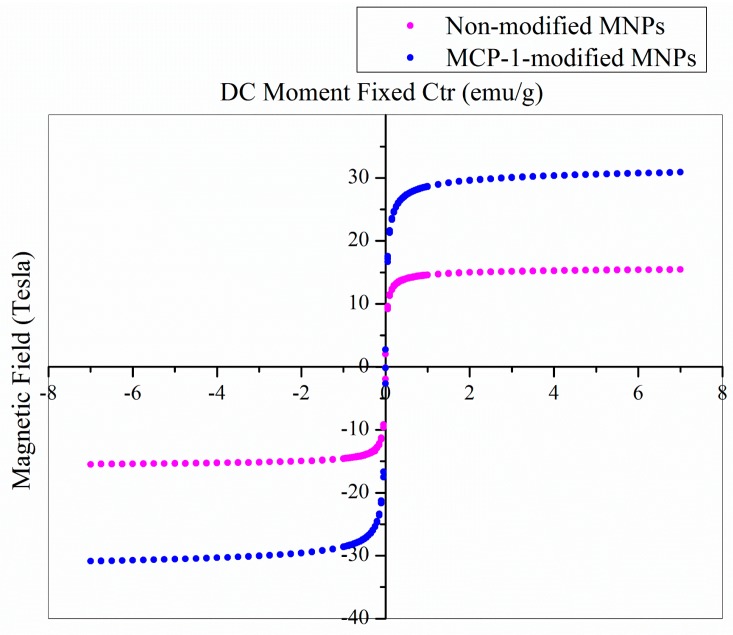
M-H curve/hysteresis loop of iron oxide MNPs.

To make sure that the magnetic properties remained after dextran coating and peptide grafting, a superconducting interference magnetometer was applied. Figure 4 shows the M-H Curve/Hysteresis Loop of MNPs (2.2 mg) and MCP-1-motif MNPs (9.0 mg). The operation temperature was 310 K and the magnetic field range was ±7.0 Tesla.

The hysteresis loops were normal and tight with no remnant magnetization, indicating a typical superparamagnetic behavior. In general, iron oxide MNPs, whose size was smaller than 20 nm, were supposed to be superparamagnetic at room temperature. Hence, from Figure 4, we could observe that the saturation magnetization values of MNPs and MCP-1-motif MNPs were 15.5 and 31.0 emu/g, respectively, due to the size difference of the two types of nanoparticles [45,46,47].

Paramagnetic materials had unpaired electrons, such as atomic or molecular orbitals. Therefore, superparamagnetic nanoparticles were free to align their magnetic moment in any direction. When an external magnetic field was applied, these magnetic moments would tend to align themselves in the same direction as the applied field. In addition, peptides derived from MCP-1 also contained free electrons and might enhance the magnetic moments in the applied magnetic field, promoting high saturation magnetization values [48].

### 3.3. Cytotoxicity

**Figure 5:**
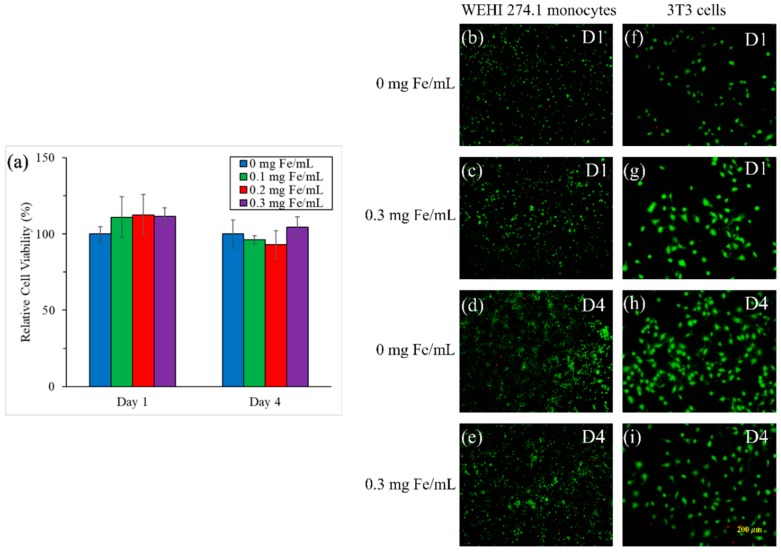
(**a**) MTT assay of 3T3 cells with different concentration of MCP-1-motif MNPs; (**b**–**i**) Live/Dead staining of WEHI 274.1 monocytes (**b**–**e**), and 3T3 cells (**f**–**i**) in 0 and 0.3 mg Fe/mL at day 1 (D1) (**b**,**c**,**f**,**g**) and day 4 (D4) (**d**,**e**,**h**,**i**) (*n* = 4).

The MTT assay was conducted with 3T3 cells incubated with different concentrations of MCP-1-motif MNPs for one day and four days. The result is shown in Figure 5a and could estimate the cytotoxicity of the nanoparticles. The normal Fe concentration of the injection solution was 0.2 mg Fe/mL. The lower and higher Fe concentrations were also investigated in the MTT assay. The percentages were calculated by comparing the 3T3 cells without iron oxide MNPs. The cytotoxicity of MCP-motif MNPs under three concentrations did not have a negative effect on cell proliferation. Even after four days of incubation, the cell viability still reached around 100%.

Figure 5b, Figure S3 and S4 are the Live/Dead staining images. The counting results are shown in Figure S5. WEHI 274.1 monocytes and 3T3 cells were also incubated with different concentrations of MCP-1-motif MNPs for one day and four days. The monocytes all maintained a round shape and the quantities were consistent with the control group. Similarly, 3T3 cells were still in elongation and proliferated stably in all groups.

### 3.4. In Vitro Imaging of MCP-1-Motif MNPs

**Figure 6:**
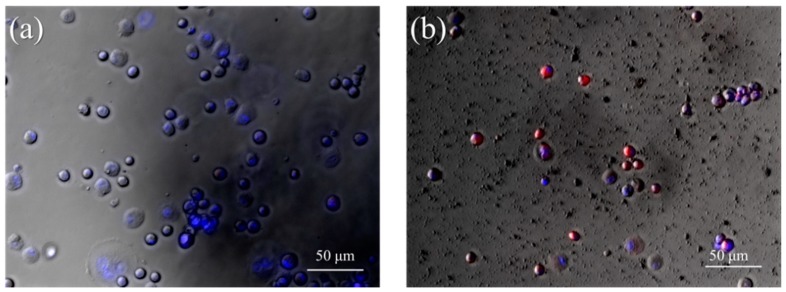
Overlaid image (fluorescence and bright) of WEHI 274.1 monocytes cultured with (**a**) Cy5-MNPs or (**b**) Cy5-MCP-1-motif MNPs.

Figure S6a is the fluorescence image of Cy5-MCP-1-motif MNPs; the further conformation can be observed through the merged image in Figure S6b. WEHI 274.1 monocytes were cultured with Cy5-MNPs or Cy5-MCP-1-motif MNPs after 1 h and stained with DAPI (Figure 6). After cultivation, the nanoparticles in solution were removed by centrifugation. Whether in the fluorescence or the bright image, there was no Cy5 signal, which represented MNPs co-localized with WEHI 274.1 monocytes. However, monocytes stained by DAPI (blue) had a spherical shape and the surfaces were overlapped with Cy5-MCP-1-motif MNPs (red), indicating the potential affinity to monocytes of peptides derived from MCP-1. Moreover, 3T3 cells were also cultured with MCP-1-motif MNPs, but no nanoparticle seemed to attach on the cell surface (Figure S7). Therefore, we could conclude that the binding ability of MCP-1 was preserved and MCP-1-motif MNPs had the ability to target monocytes.

### 3.5. Nuclear Magnetic Resonance Imaging (MRI)

**Figure 7:**
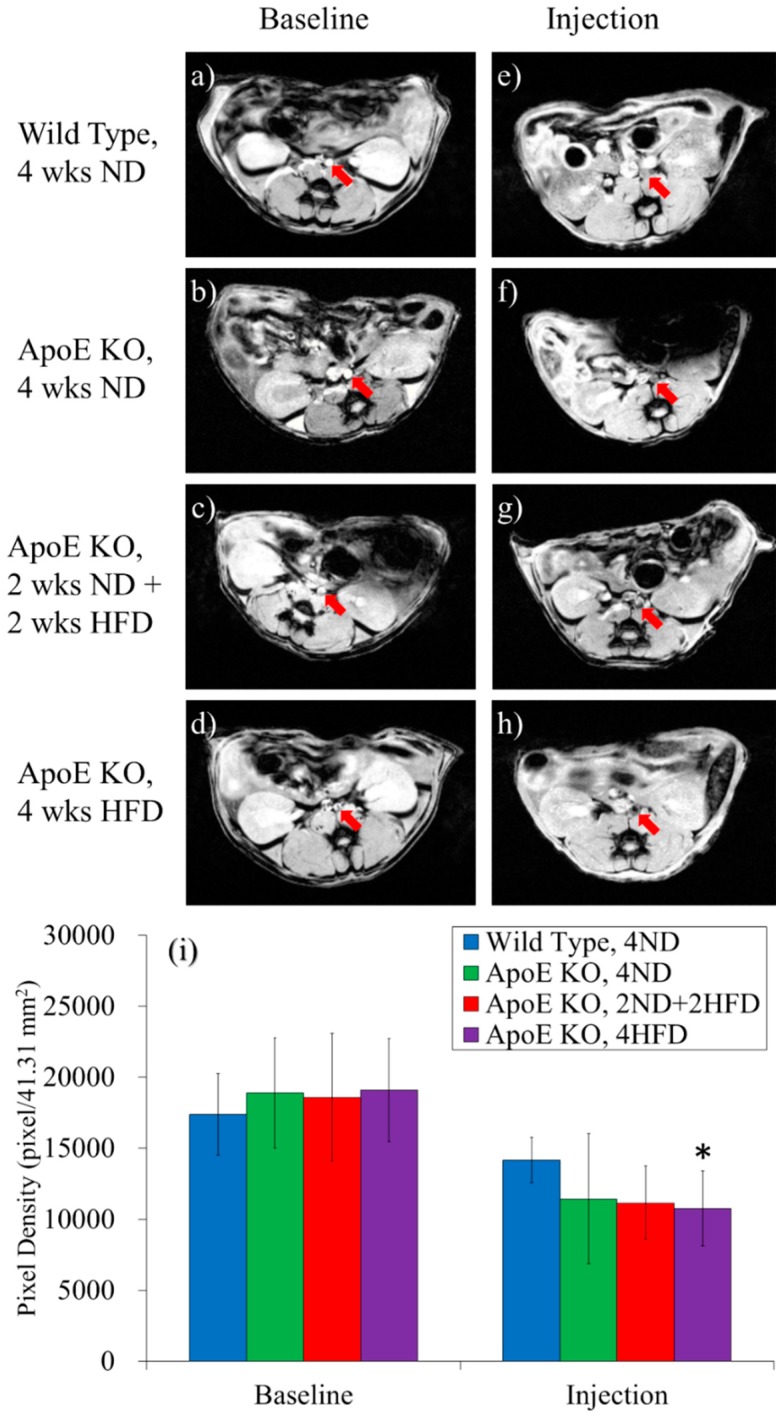
(**a**–**h**) Magnetic resonance images of mice injected with MCP-1-motif MNPs before and after experiments (wks = weeks); (**i**) Diagram of pixel density throughout the aorta area (*n* = 3) (* *p* < 0.05, compared to the same group of baseline).

Magnetic resonance microscopy permitted to us obtain high-resolution images of the aorta in mice at the level of the abdominal aorta. Figure 7a–h show the MRI abdomen axial cross-sectional anatomy of wild-type mice and ApoE KO mice. The upper side of the images is anterior and the lower side is posterior. Before the nanoparticle injection, all mice were scanned for baseline, which are recorded in Figure 7a–d. The red arrowhead symbol indicates the abdominal aortic walls of mice and the light color represents the hollow structure.

Figure 7e–h are the images of magnetic resonance images of mice injected with MCP-1-motif MNPs after 40 h. Bright aortic lumen and wall indicated that there was no significant iron oxide MNPs accumulation in wild-type mice (Figure 7e). Besides, ApoE KO mice with four weeks of high-fat diet had dark aorta walls, as shown in Figure 7h, indicating that the aorta was full of MCP-1-motif MNPs. The degree of darkness in the aorta was more obvious when mice were fed a high-fat diet for a longer period of time [49,50,51].

Figure 7i shows the pixel density of aorta (area = 41.31 mm^2^), which stood for the degree of light color. The values of pixel density of all groups decreased because of the existence of iron oxide MNPs. The ratio of pixels decreased after injections: 25.43% (wild-type mice ND4), 26.51% (ApoE KO mice ND4), 38.14% (ApoE KO mice ND2 + HFD2), 40.86% (ApoE KO mice HFD4). This indicated that MCP-1-motif MNPs in the blood flow obviously attached to the monocytes during the formation of atherosclerosis plaques in ApoE mice compared to the other three groups. The MRI results concluded that the MCP-1-motif MNPs would accumulate in the aorta in the atherosclerosis model.

### 3.6. Non-Invasive In Vivo Imaging System

**Figure 8:**
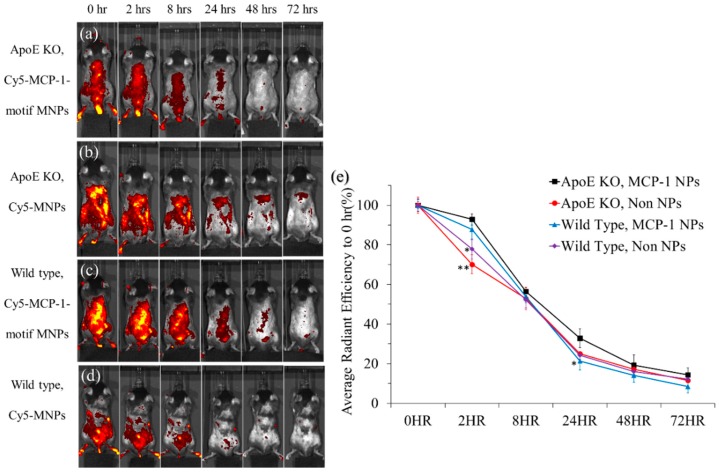
(**a**–**d**) IVIS body fluorescence of ApoE KO mice, wild-type mice injected with Cy5-MCP-1-motif MNPs and Cy5-MNPs; (**e**) average radiant efficiency at 0 h of IVIS body fluorescence (*n* = 3) (* *p* < 0.05 compared with ApoE KO, MCP-1 NPs at the same injection time; ** *p* < 0.01 compared with ApoE KO, MCP-1 NPs at the same injection time).

The IVIS spectrum system confirmed the existence of Cy5 MNPs and Cy5-MCP-1-motif MNPs in the bloodstream of mice in 72 h through a background level of fluorescence throughout the body. Figure 8 and Figure S8 show the body fluorescence images of ApoE mice as well as wild-type mice injected with iron oxide MNPs, respectively. Body fluorescence decreased due to nanoparticles being excreted when the time reached 48 or 72 h. The fluorescence distribution in MCP-1-motif MNPs (Figure 8) tended to accumulate at the backbone of the body, which might indicate the aggregation of iron oxide MNPs in the aorta. Compared to the experimental groups, MNPs distributed randomly throughout the body.

Figure 8e shows the backbone fluorescence at any time point compared to the 0-h backbone fluorescence. The fluorescence of ApoE KO mice with MCP-1-motif MNPs still maintained over 90% at 2 h compared to the other test groups. On the other hand, the fluorescence of MNPs in the two types of mice all reduced to under 80% at 2 h, and the two wild-type groups were significantly different from the ApoE KO mice with the injection of MCP-1-motif MNPs. This might result from the retention ability of MCP-1-motif MNPs in the atherosclerosis model. After the 24-h injection, the signals all decreased to about 30–40%, and they declined to under 20% in 72 h [52].

**Figure 9:**
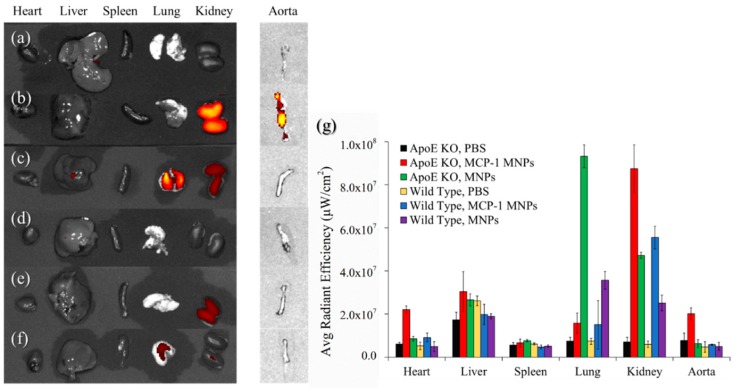
IVIS organ fluorescence of ApoE KO mice injected with (**a**) PBS; (**b**) MCP-1-motif MNPs; (**c**) MNPs, and wild-type mice injected with (**d**) PBS; (**e**) MCP-1-motif MNPs; (**f**) MNPs. (**g**) Average radiant efficiency diagram of IVIS organ fluorescence (*n* = 3).

After the 72-h injection, mice were sacrificed and the aorta, heart, liver, spleen, lung, and kidney were harvested. The aorta and organs fluorescence conducted by IVIS are shown in Figure 9. From the results of different organs, the presence of iron oxide MNPs was detected in the kidney of all types of mice, indicating that mice would excrete the nanoparticles in urine through the kidney and the bladder. However, the aorta of the atherosclerosis model injected with MCP-1-motif MNPs had notable fluorescence signals, confirming the longer retention time of MCP-1-motif MNPs to atherosclerosis plaque in the aorta.

### 3.7. Iron and Oil Drops in Specific Organs

**Figure 10:**
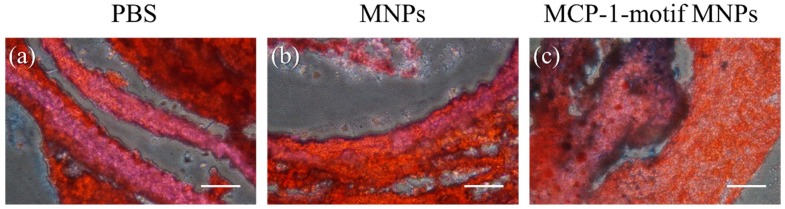
Prussian Blue and Oil Red O staining of the aorta of ApoE KO mice with injection of (**a**) PBS, (**b**)MNPs, and (**c**) MCP-1-motif MNPs (scale bar = 50 μm).

After in vivo imaging, kidneys were cut into 6–8 μm cross-sections and the tissue sections were stained with hematoxylin and Prussian Blue (Figure S9). Blue precipitation found in the kidney tissue due to the injection of iron oxide MNPs confirmed that the kidney was the major organ employed for excreting nanoparticles [31]. For further confirmation of MCP-1-motif MNPs in the atherosclerosis model, the vessel wall of aorta was stained with Oil Red O, which illustrated atherosclerotic lesions, and Prussian Blue, which illustrated precipitations of iron oxide MNPs (Figure 10). The staining results showed the co-localization of MCP-1-motif MNPs and plaques. Oil drops and Prussian Blue are all observed in Figure 10c, indicating the potential affinity of MCP-1-motif MNPs in the atherosclerosis model [53,54,55].

## 4. Conclusions

In this work, the characteristics of iron oxide MNPs were measured and analyzed. Electron microscope images showed the quasi-spherical shape of nanoparticles and XPS spectra further confirmed that the MCP-1 peptides conjugated on the iron oxide MNPs. They were relatively stable in aqueous solution and dispersive when stirred.

Then, the following in vitro experiment was examined. We developed the cell viability process of iron oxide MNPs, including an MTT assay and a Live/Dead assay. Whether in WEHI 274.1 monocytes or 3T3 cells, MCP-1-motif MNPs exhibited a cell viability around 100% compared to the control group. Next, we successfully attached Cy5 fluorescence to iron oxide MNPs using an amine-ester reaction. With different types of cells incubated with iron oxide MNPs for 1 h, we observed that MCP-1-motif MNPs co-localized with WEHI 274.1 monocytes, indicating a potential tool for tracking early-stage atherosclerosis lesions in aorta, usually aggregated with large quantities of monocytes.

Next, iron oxide MNPs were injected into wild-type mice, for the control group, and ApoE KO mice, a widely-used atherosclerosis model, and in vivo imaging was performed. First, MRI showed that MCP-1-motif MNPs were obviously accumulated in the abdomen aorta of ApoE KO mice fed a high-fat diet for four weeks compared to other experimental groups. Second, we also found that the average radiant efficiency of Cy5-MCP-1-motif MNPs nearby the backbone of the atherosclerosis model mice was still 90% after 2 h. In addition, organs harvested after 72 h showed that nanoparticles accumulated in the kidney for excretion in all mice injected with nanoparticles. Above all, Cy5-MCP-1-motif MNPs largely aggregated in the aorta of atherosclerosis model mice, confirming the co-localization of aorta plaque and MCP-1-motif MNPs. Finally, vital organs tissues were stained with Prussian Blue and Oil Red O, which identified that the MCP-1-motif MNPs have the potential ability to track aorta lesions and can be a promising targeting tool for early-stage atherosclerosis.

In conclusion, from the in vitro test and in vivo test, we observed that MCP-1-motif MNPs could interact with monocytes and accumulate in the aorta of an atherosclerosis model, indicating a potential targeting tool for early-stage atherosclerosis.

## Figures and Tables

**Table 1:** Experimental design of animal model for magnetic resonance imaging (MRI).

Mice	Diet	Nanoparticle Injection
Wild-type	Four weeks ND	MCP-1-motif MNPs
ApoE KO	Four weeks ND	MCP-1-motif MNPs
ApoE KO	Two weeks ND and two weeks HFD	MCP-1-motif MNPs
ApoE KO	Four weeks HFD	MCP-1-motif MNPs

Normal diet (ND), High fat diet (HFD), monocytes chemoattractant protein-1 (MCP-1), magnetic nanoparticles (MNP).

**Table 2:** Experimental design of animal model for in vivo imaging system (IVIS).

Mice	Diet	Nanoparticle Injection
Wild-type	Four weeks ND	PBS
Wild-type	Four weeks ND	MNPs
Wild-type	Four weeks ND	MCP-1-motif MNPs
ApoE KO	Four weeks HFD	PBS
ApoE KO	Four weeks HFD	MNPs
ApoE KO	Four weeks HFD	MCP-1-motif MNPs

**Table 3:** Dynamic light scattering (DLS) results of MNPs and MCP-1-motif MNPs (*n* = 3).

Nanoparticles	Zeta Potential (mV)	Hydrodynamic Diameter (nm)
MNPs	−14.1 ± 0.16	90.0 ± 4.90
MCP-1-motif MNPs	−17.6 ± 0.25	323.8 ± 12.17

**Table 4:** Chemical element composition ratio of iron oxide MNPs.

	MNPs	MCP-1-Motif MNPs
C1s	54.25%	40.32%
N1s	5.44%	2.14%
O1s	40.31%	57.55%

## Supplementary Materials

The following are available online at http://www.mdpi.com/1999-4923/10/2/62/s1, Figure S1: Characterization of (a) MNPs and (b) MCP-1-motif MNPs using SEM, Figure S2: Hydrodynamic distribution of (a) MNPs and (b) MCP-1-motif MNPs by DLS, Figure S3: Live/Dead assay of cells with MCP-1-motif MNPs at day 1, Figure S4: Live/Dead assay of cells with MCP-1-motif MNPs at day 4, Figure S5: Cell counting of (a) WEHI 274.1 monocytes and (b) 3T3 cells. (*n* = 3), Figure S6: (a) Fluorescence image and (b) merged image of the Cy5-MCP-1-motif MNPs, Figure S7: Fluorescence image of (a) 3T3 cells and (b) WEHI 274.1 monocytes cultured with Cy5-MCP-1-motif MNPs, Figure S8: IVIS body fluorescence of ApoE KO mice and wild-type mice injected with PBS from 0- to 72-h injection, Figure S9: Prussian Blue and hematoxylin staining of kidney of (a–c) ApoE KO mice and (d–f) wild-type mice (scale bar = 50 μm), Table S1. Bond composition ratios of the iron oxide MNPs.
