# Supplementary material for: Magnetic Nanoparticles Conjugated with Peptides Derived from Monocyte Chemoattractant Protein-1 as a Tool for Targeting Atherosclerosis
Source: Pharmaceutics. 2018 May 24;10(2):62. doi: 10.3390/pharmaceutics10020062 (PMC6027309; doi:10.3390/pharmaceutics10020062)
Supplement: Supplementary file 1 [file pharmaceutics-10-00062-s001.pdf]

Supplementary Materials: Magnetic Nanoparticles Conjugated with Peptides Derived from Monocyte Chemoattractant Protein-1 as a Tool for Targeting Atherosclerosis

Chung-Wei Kao, Po-Ting Wu, Mei-Yi Liao, I-Ju Chung, Kai-Chien Yang, Wen-Yih Isaac Tseng, Jiashing Yu

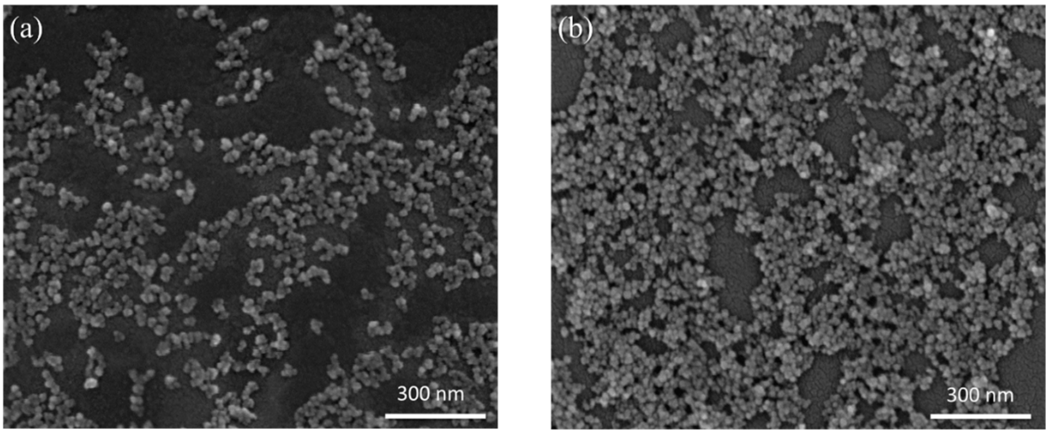

Figure S1. Characterization of (**a**) magnetic nanoparticles (MNPs) and (**b**) monocytes chemoattractant protein-1 (MCP-1)-motif MNPs using scanning electron microscope (SEM).

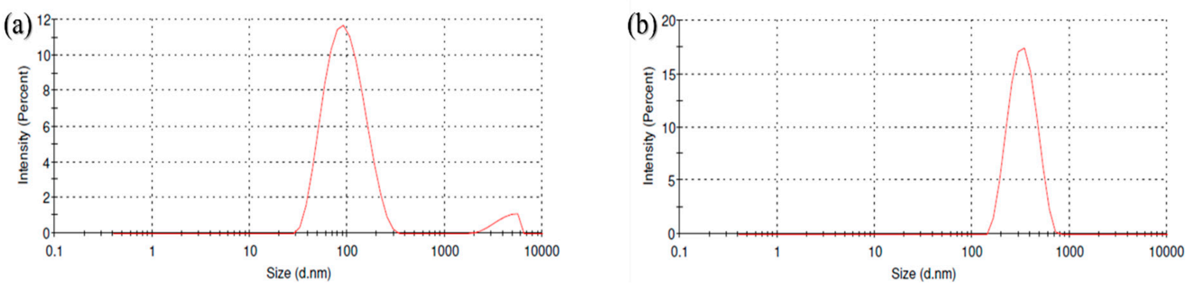

Figure S2. Hydrodynamic distribution of (**a**)MNPs and (**b**)MCP-1-motif MNPs by dynamic light scattering (DLS).

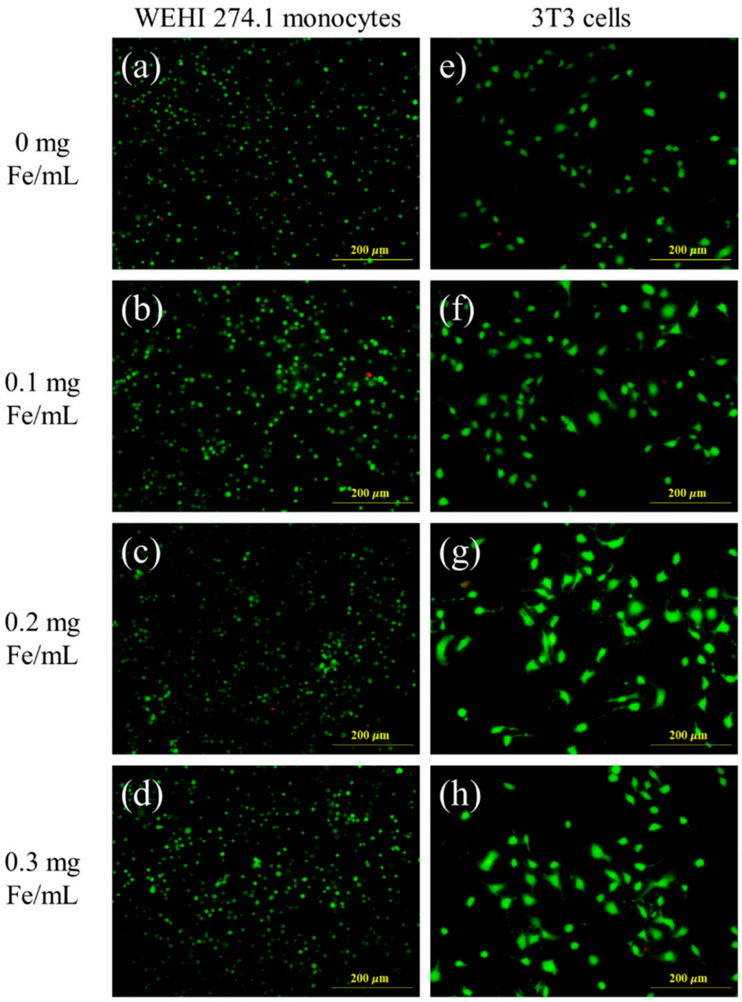

Figure S3. Live/Dead assay of (**a**–**d**) WEHI 274.1 monocytes and (**e**–**h**) 3T3 cells s with 0 to 0.3 mg Fe/mL MCP-1-motif MNPs at day 1.

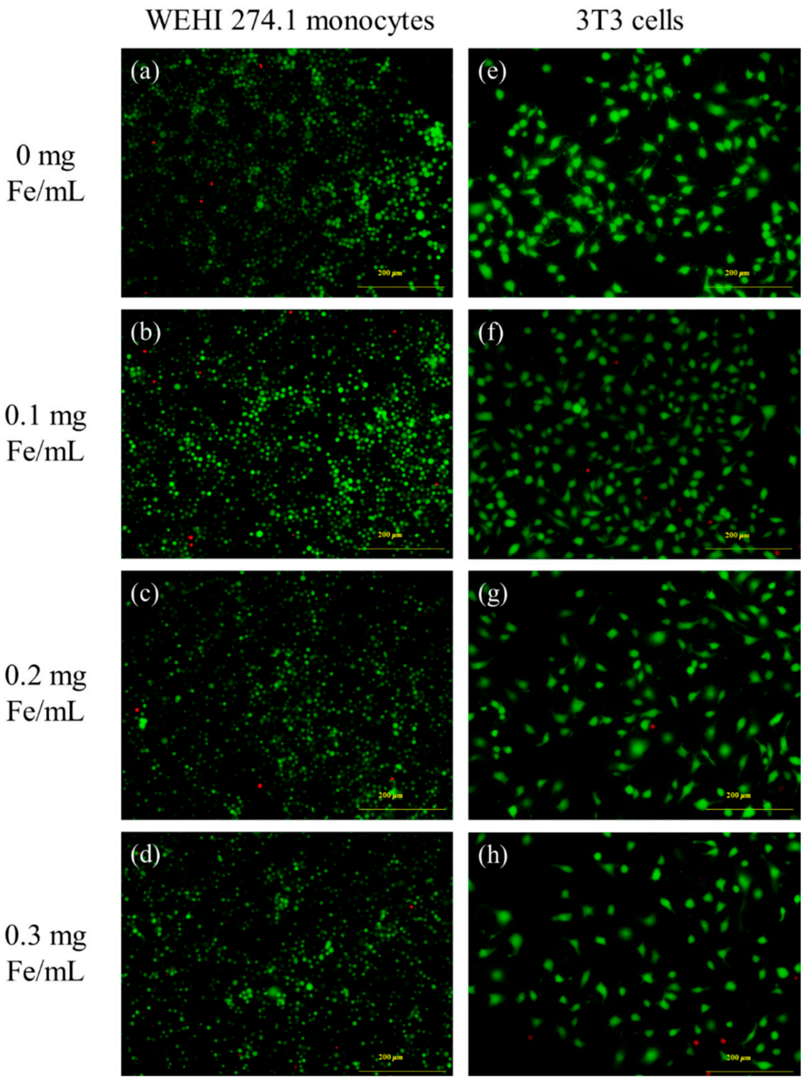

Figure S4. Live/Dead assay of (**a**–**d**) WEHI 274.1 monocytes and (**e**–**h**) 3T3 cells s with 0 to 0.3 mg Fe/mL with MCP-1-motif MNPs at day 4.

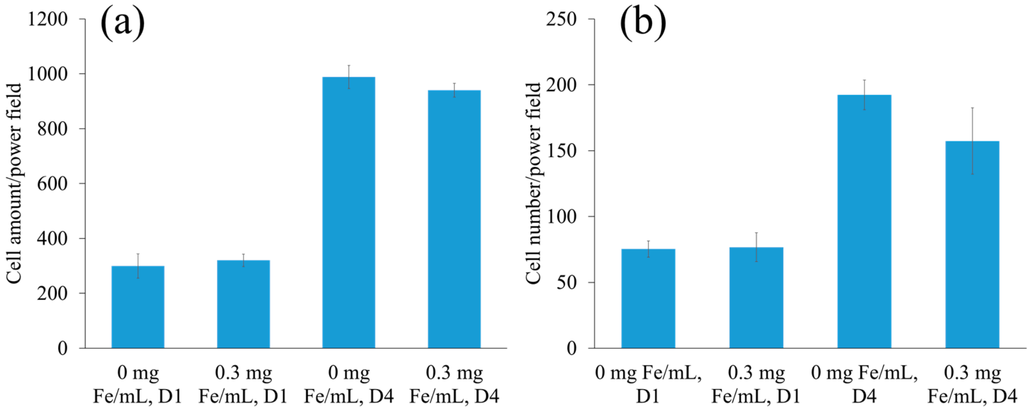

Figure S5. Cell counting of (**a**) WEHI 274.1 monocytes and (**b**) 3T3 cells. (n = 3).

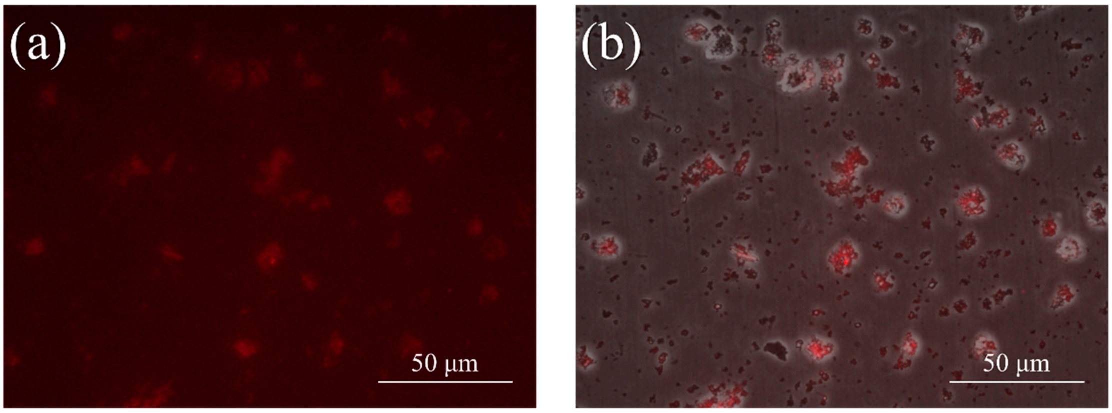

Figure S6. (**a**) Fluorescence image and (**b**) optical image of the Cy5-MCP-1-motif MNPs.

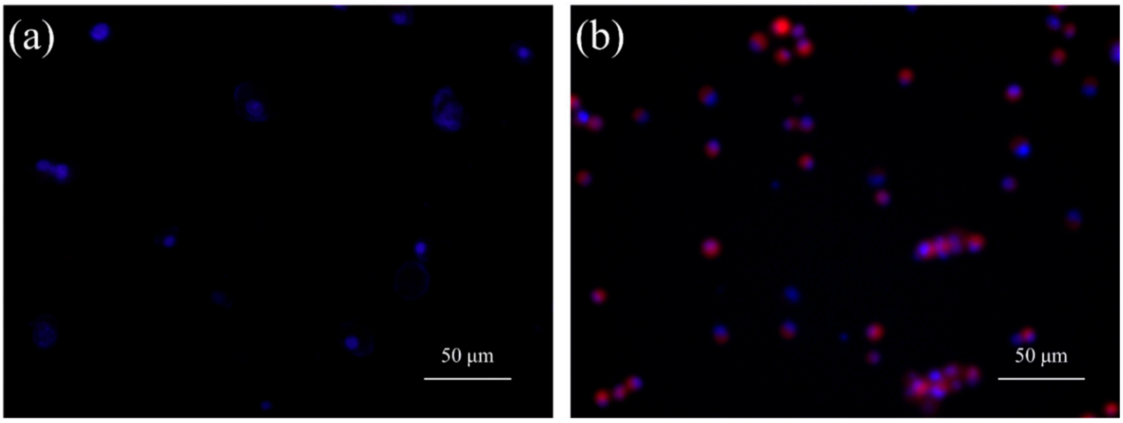

Figure S7. Fluorescence image of (**a**) 3T3 cells and (**b**) WEHI 274.1 monocytes cultured with Cy5-MCP-1-motif MNPs.

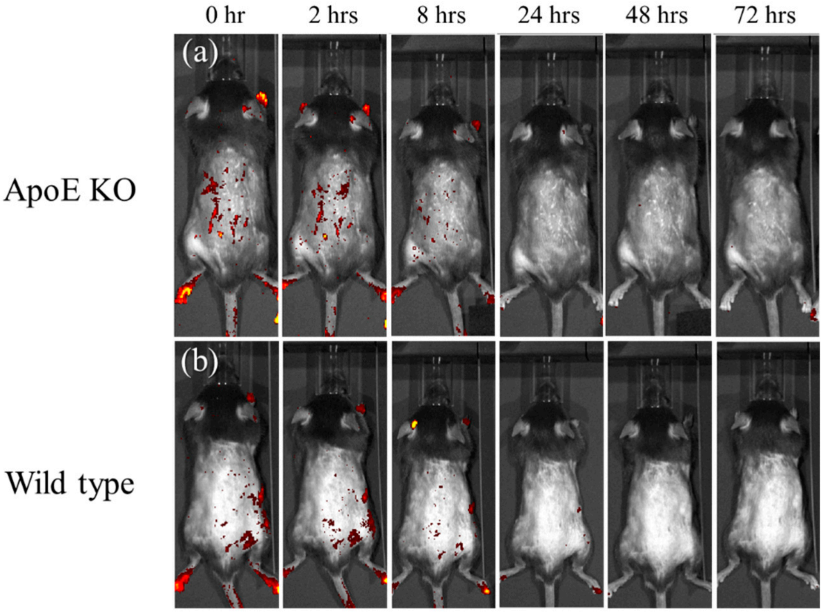

Figure S8. IVIS body fluorescence of (**a**) ApoE KO mice and (**b**) wild type mice injected with PBS from 0 to 72 hr-injection.

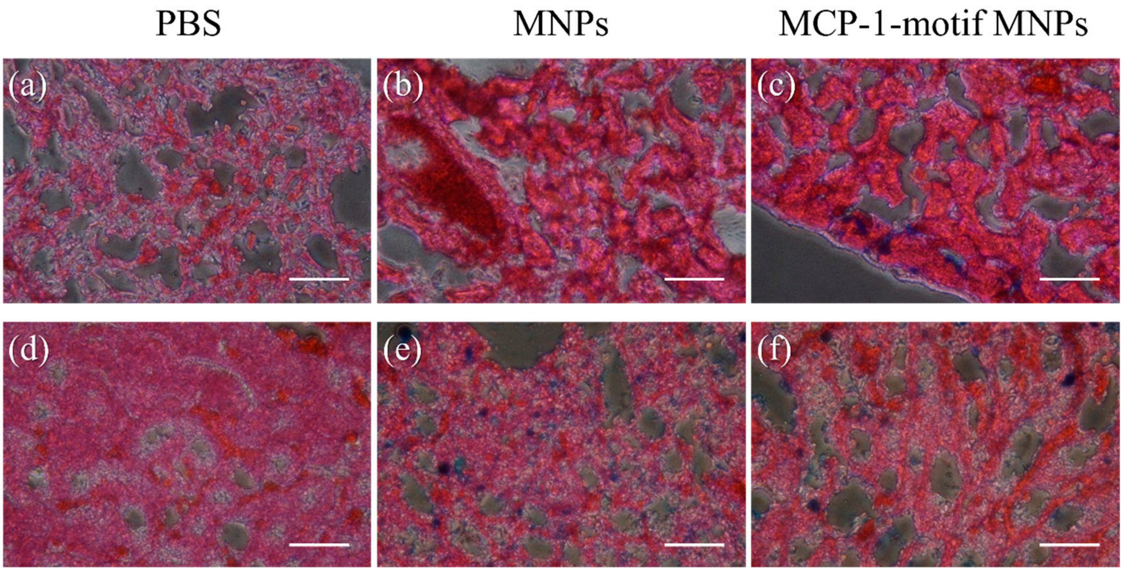

Figure S9. Prussian Blue and hematoxylin staining of kidney of (**a**–**c**) ApoE KO mice and (**d**–**f**) wild type mice (scale bar = 50 μm).

Table S1. Bond composition ratio of the iron oxide MNPs.

	Bond	Binding Energy (eV)	MNPs	MCP-1-motif MNPs
			Percentage (%)	
**C1s**	C-C	284.5	99.9	77.2
	C-O	286.0	~0.1	19.5
	C=O	288.0	~0.1	3.3
**N1s**	N(H)-C	398.8	99.7	20.0
	N(C)-C	400.0	0.2	80.0
**O1s**	O=C	532.0	17.6	57.7
	O-C	533.3	~0.1	2.0
	Fe_3O_4	529.7	31.5	22.0
	H_2O	535.3	50.9	18.3

Magnetic nanoparticles (MNPs), monocytes chemoattractant protein-1 (MCP-1), carbon 1s orbital (C1s), nitrogen 1s orbital (N1s), oxygen 1s orbital (O1s).
